# De novo variants underlying monogenic syndromes with intellectual disability in a neurodevelopmental cohort from India

**DOI:** 10.1038/s41431-023-01513-7

**Published:** 2023-12-20

**Authors:** Shruti Pande, Purvi Majethia, Karthik Nair, Lakshmi Priya Rao, Selinda Mascarenhas, Namanpreet Kaur, Michelle C. do Rosario, Kausthubham Neethukrishna, Ankur Chaurasia, Bhagesh Hunakunti, Nalesh Jadhav, Sruthy Xavier, Jeevan Kumar, Vivekananda Bhat, Gandham SriLakshmi Bhavani, Dhanya Lakshmi Narayanan, B.L. Yatheesha, Siddaramappa J. Patil, Sheela Nampoothiri, Nutan Kamath, Shrikiran Aroor, Y Ramesh Bhat, Leslie E. Lewis, Suvasini Sharma, Shruti Bajaj, Naveen Sankhyan, Shahyan Siddiqui, Shalini S. Nayak, Stephanie Bielas, Katta Mohan Girisha, Anju Shukla

**Affiliations:** 1 Department of Medical Genetics, Kasturba Medical College, Manipal, Manipal Academy of Higher Education, Manipal, India; 2 Division of Evolution, Infection and Genomics, School of Biological Sciences, Faculty of Biology, Medicine and Health, University of Manchester, Manchester M13 9PL, United Kingdom; 3 Dheemahi Child Neurology and Development Center, Shivamogga, India; 4 Division of Medical Genetics, Mazumdar Shaw Medical Center, Narayana Hrudayalaya Hospitals, Bangalore, India; 5 Department of Pediatric Genetics, Amrita Institute of Medical Sciences & Research Centre, Cochin, India; 6 Department of Paediatrics, Kasturba Medical College, Mangalore, Manipal Academy of Higher Education, Manipal, India; 7 Department of Paediatrics, Kasturba Medical College, Manipal, Manipal Academy of Higher Education, Manipal, India; 8 Neurology Division, Department of Pediatrics, Lady Hardinge Medical College and Associated Kalawati Saran Children’s Hospital, New Delhi, India; 9 The Purple Gene clinic, Mumbai, India; 10 Pediatric Neurology Unit, Department of Pediatrics, Advanced Pediatrics Centre, Postgraduate Institute of Medical Education & Research, Chandigarh, India; 11 Department of Neuro and Vascular Interventional Radiology, Yashoda Hospitals, Secunderabad, Hyderabad, India; 12 Department of Human Genetics, University of Michigan, Ann Arbor, MI, United States of America; 13 Suma Genomics Private Limited, Manipal Center for Biotherapeutics Research, Manipal Academy of Higher Education, Manipal, India; 14 Department of Genetics, College of Medicine & Health Sciences, Sultan Qaboos University, Muscat, Oman

## Abstract

The contribution of de novo variants as a cause of intellectual disability (ID) is well established in several cohorts reported from the developed world. However, the genetic landscape as well as the appropriate testing strategies for identification of de novo variants of these disorders remain largely unknown in low-and middle-income countries like India. In this study, we delineate the clinical and genotypic spectrum of 54 families (55 individuals) with syndromic ID harboring rare de novo variants. We also emphasize on the effectiveness of singleton exome sequencing as a valuable tool for diagnosing these disorders in resource limited settings. Overall, 46 distinct disorders were identified encompassing 46 genes with 51 single-nucleotide variants and/or indels and two copy-number variants. Pathogenic variants were identified in *CREBBP, TSC2, KMT2D, MECP2, IDS, NIPBL, NSD1, RIT1, SOX10, BRWD3, FOXG1, BCL11A, KDM6B, KDM5C, SETD5, QRICH1, DCX, SMARCD1, ASXL1, ASXL3, AKT3, FBN2, TCF12, WASF1, BRAF, SMARCA4, SMARCA2, TUBG1, KMT2A, CTNNB1, DLG4, MEIS2, GATAD2B, FBXW7, ANKRD11, ARID1B, DYNC1H1, HIVEP2, NEXMIF, ZBTB18, SETD1B, DYRK1A, SRCAP, CASK, L1CAM*, and *KRAS*. Twenty-four of these monogenic disorders have not been previously reported in the Indian population. Notably, 39 out of 53 (74%) disease-causing variants are novel. These variants were identified in the genes mainly encoding transcriptional and chromatin regulators, serine threonine kinases, lysosomal enzymes, molecular motors, synaptic proteins, neuronal migration machinery, adhesion molecules, structural proteins and signaling molecules.

## Introduction

Intellectual disability (ID) is defined as a defect in cognitive functioning and adaptive behavior that originates before the age of 18 years and has a worldwide prevalence of ~2−3% [1]. The etiology of ID includes both acquired as well as genetic causes [2, 3]. The genetic etiology of disorders of ID is highly heterogeneous, encompassing a wide spectrum of genetic variations, including structural variants (SVs), copy number variants (CNVs), small insertions/deletions, and single-nucleotide variants (SNVs), identified across more than 1000 genes [4–7]. Monogenic disorders contribute to 30−50% of cases of ID, around 20% are due to disease-causing large or small CNVs and the cause of ~50% cases remains unknown till date [4, 5, 8, 9].

Disorders which have a component of ID and are associated with other systemic or behavioral abnormalities are referred to as syndromic ID (sID). These disorders follow all inheritance patterns and show extreme genetic heterogeneity. With the availability of rapidly advancing next generation sequencing (NGS) based platforms, mainly exome and genome sequencing (ES/GS) has led to the rapid and efficient diagnosis as well as discovery of several ID syndromes in the last two decades [10].

De novo variants are now a well-recognized cause of severe early-onset genetic diseases, including sIDs. Though the genetic basis of inherited sIDs could be identified through studies involving large family pedigrees, the sporadic ones remained largely unidentified until recently. NGS approaches, particularly proband-parents trio ES/GS is an effective method of understanding the distribution of variations and determining all types of de novo events throughout the genome, from SNVs, indels, CNVs to large SVs in addition to determining the parental origin and whether they occurred in the germline or post zygotically [4, 8, 9, 11].

The challenges faced by the low- and middle-income countries (LMIC) in terms of rare disease diagnosis using the evolving genomic technologies include the lack of coherent national policies, limited trained professionals, lagging research infrastructure, and lastly economic and cultural challenges [12, 13]. The extent of the burden posed by de novo variants associated with ID syndromes remains incompletely understood within several LMICs, including India. With the aim to address this challenge, we herein represent the clinical and genetic spectrum of 54 families with de novo variants underlying sID. We also highlight the utility of proband-only ES followed by segregation analysis as a first-tier testing in identification of de novo variants in resource-limited settings.

## Material And Methods

We evaluated and recruited 530 families with heterogeneous neurodevelopmental disorders (NDDs) in an ongoing mono centric study from October 2019 to December 2022. The clinical characteristics of the affected individuals were recorded through detailed clinical examination using human phenotype ontology (HPO terms). Informed consents for genetic testing, publication of data and clinical photographs were obtained from the families. The informed consents were approved by the Institutional ethics committee, Kasturba Medical College and Kasturba Hospital, India as per the declaration of Helsinki.

Genomic DNA was extracted from the peripheral blood sample of the proband, parents and siblings (as required) using the QIAamp DNA Blood Mini Kit (QIAGEN, Valencia, CA; cat # 51106). The testing strategy included either an exome first or a sequential testing approach in which a targeted test or chromosomal microarray (CMA) was followed by ES for the affected individuals based on the clinical phenotype. The NGS data processing, quality assessment, variant calling, annotation and analysis was performed as described earlier [14]. The Sanger validation and segregation analysis was carried out in all families with a singleton ES or Mendeliome while Sanger validation was carried out in families who achieved a diagnosis using a trio ES. CNV analysis from exome data was performed for individuals with no clinically relevant SNVs/indels detected on ES. The detailed description of Mendeliome, CNV and ES analysis is provided in the supplementary material.

## Results

Of the 530 families recruited, 211 affected individuals from 196 families presented with a syndromic presentation characterized by major or minor morphologic anomalies and neurologic, cognitive, behavioral or sensory impairments. A molecular diagnosis could be achieved in 104 of the total 196 families (53%). Of these, 59 families (57%) carried de novo variants, six families (4%) had inherited variants underlying an autosomal dominant or an X-linked disorder, 16 families (16%) had biallelic variants underlying an autosomal recessive disorder, and 23 families (23%) were diagnosed with a chromosomal aberration. Consanguinity was noted in 25 families (24%). Within the 25 consanguineous families, 14 families (56%) had biallelic variants underlying an autosomal recessive disorder, two families (8%) had inherited variants underlying X-linked recessive disorders, six (24%) carried de novo variants causing autosomal dominant and X-linked dominant disorders, and three families (12%) had CNVs.

**Fig. 1:**
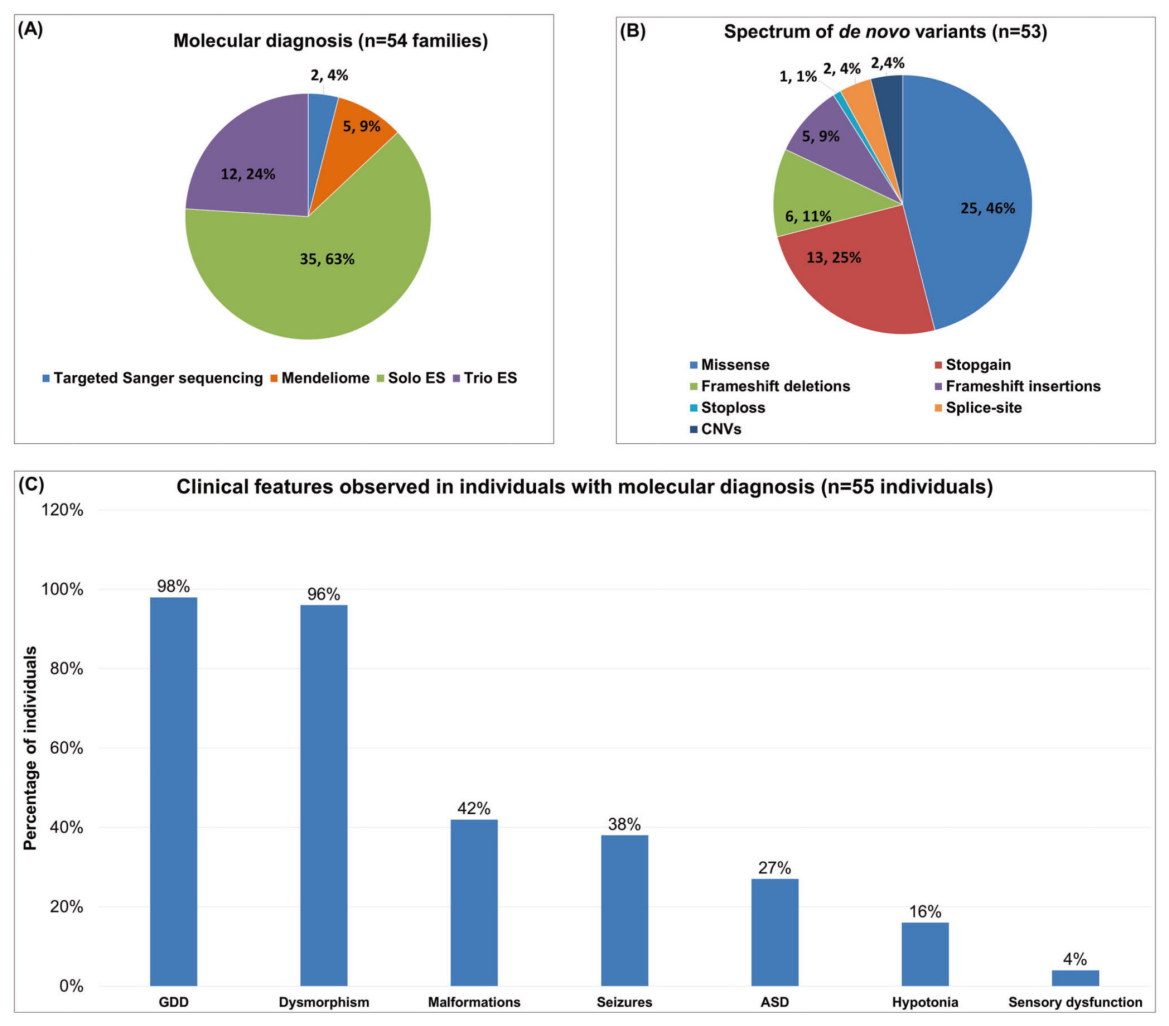
Cohort details depicting. **A** Types of genetic testing employed **B** Disease-causing de novo variants identified **C** Spectrum of the clinical findings observed in individuals with sID. GDD Global developmental delay, ASD Autism spectrum disorder.

Of the 59 families with de novo variants, five families (five affected individuals) with novel disease-gene association, phenotypic expansion, and multiple genetic diagnoses have been published earlier [15–17]. The present cohort consists of 55 individuals from 54 families diagnosed using targeted Sanger sequencing, Mendeliome, singleton ES, and trio ES (Fig. 1A and Table 1). Thirty-one affected individuals were males (57%) and 24 were females (43%). Consanguinity was noted in six families (11%). The age ranged from newborn to 14 years. The clinical findings noted in 55 diagnosed individuals, in addition to ID, included global developmental delay, dysmorphism, malformations, seizures, autism spectrum disorder, hypotonia, and sensory dysfunction (Fig. 1C). A total of 53 disease-causing de novo variants underlying 46 distinct ID syndromes were identified in the current cohort (Table 1). Of which, 51 were SNVs and/or indels and two were CNVs (Fig. 1B). Notably, 39 (74%) of them were found to be novel. These SNVs and indels were classified according to the American College of Medical Genetics and Genomics (ACMG) and the Association for Molecular Pathology (AMP) standards and guidelines for interpreting sequence variants [18]. Thirty variants were classified as pathogenic (58%) and 21 as likely pathogenic (40%). The two CNVs identified were classified as pathogenic according to the American College of Medical Genetics and Genomics and ClinGen standards and guidelines for CNVs (Table 1) [19]. The additional details pertaining to the genetic testing performed, disease-causing variants, and ClinVar submission IDs are provided in Supplementary Table S1.

## Discussion

Several cohorts of individuals harboring disease-causing de novo variants and underlying sIDs have been reported in the last two decades. However, most of these cohorts have originated from Caucasian outbred populations of the developed world [20–22]. The spectrum as well as the burden of the variants contributing to sID in LMICs remains largely uninvestigated. The current study elucidates the clinical and genotypic spectrum of 54 families with de novo variants underlying sID in an Indian cohort of neurodevelopmental disorders. Though several sIDs are clinically recognizable and less challenging to diagnose than isolated ID, they are often associated with large variability in the phenotypes especially among different populations. This variability could be because of a difference in genetic background, environmental factors or a combination of both. In addition to ID, global developmental delay, dysmorphism, malformations, seizures, autism spectrum disorder, hypotonia and sensory dysfunction defects were the other more commonly observed comorbidities. Of the 46 disorders observed in this cohort, 24 disorders are being reported through our cohort for the first time in the Indian population, to the best of our knowledge.

Genetics of monogenic sID is extremely heterogeneous and follows all inheritance patterns. Rare de novo variants are known to contribute to causative variants in 40–70% individuals with ID [9, 21, 22]. Despite the recently published large cohort studies, the precise burden of de novo variants remains largely unknown. In the present study, we noted that 57% (54 families) of the 104 molecularly diagnosed families with sID carried de novo variants for autosomal dominant or X-linked disorders. Thirty-nine of the 53 (74%) disease-causing variants identified were novel, thus expanding the list of the disease-causing variants in sID causative genes. All these disease-causing variants were submitted to ClinVar to make them available to the medical genetics’ community worldwide.

Until recently, CMA was recommended as a first-tier test for investigating undiagnosed disorders of ID and congenital anomalies with diagnostic yields ranging from 16 to 28% [23, 24]. However, currently it is recommended that ES/GS which can identify the genetic etiology in 28–68% individuals be strongly considered as a first- or second-tier test [25, 26]. Previous studies have mainly utilized trio ES as a first-tier test to identify de novo variants in sIDs, resulting in a high diagnostic yield of 50–70% [9, 21, 22, 27]. In the current study, it was observed that 72% of the de novo variants could be identified using a singleton ES/Mendeliome. This can be attributed to additional phenotypic clues in sID aiding the interpretation of the singleton ES data and reaffirms the findings previously observed in Indian studies highlighting the effectiveness of deep phenotyping and singleton ES in diagnosis of clinically heterogeneous NDDs [13, 28, 29]. However, in families with isolated ID, variant prioritization often warrants familial testing through trio exome sequencing and/or testing of additional affected and unaffected family members. Moreover, the overall diagnostic yield of identifying monogenic de novo variants has increased from 55% (SNVs/indels) to 57% using a combinatorial approach of detecting SNVs/indels and CNVs from the exome data. These results are in line with previous studies highlighting the significance of incorporating exome based CNV analysis algorithms to increase the diagnostic yield of NDDs [30, 31].

Previous studies on syndromic/nonsyndromic ID cohort of de novo variations showed that genes encoding transcriptional and chromatin regulators were the most commonly mutated genes as compared to other neuronal regulators (synaptic maintenance and signaling) or fundamental cellular processes (translation, cell cycle control and energy metabolism) regulating genes [27, 32, 33]. In this study, we classified the genes carrying the disease-causing de novo variants in the current cohort based on its function, and our results were similar to those observed by Taskiran et al (2021). We observed that the transcriptional and chromatin regulators represented the largest class of ID-associated genes, followed by signaling molecules, serine threonine kinases, neuronal migration machinery, structural proteins, molecular motors, synaptic proteins, adhesion molecules, enzymes, microtubule formation, and cell proliferation (Supplementary Fig. 1).

It is known that approximately 80% of all de novo germline single nucleotide variants arise on the paternal allele [11, 34]. Thus, advanced paternal age at conception has been established as the major factor linked to the increase in the number of de novo variants, a subset of which might underlie developmental disorders [8, 11, 35]. However, we noted that in our cohort, the median paternal age at the time of conception was 39 years in families with autosomal recessive disorders, 29 years for inherited autosomal dominant/X-linked disorders, and 35 years in families with de novo variants.

Consanguinity and inbreeding are widely prevalent in specific communities and geographic regions of India [14, 36]. Though, the high rate of parental consanguinity and inbreeding is expected to precipitate rare autosomal recessive disorders including those causative of sIDs, we observed de novo variants in 24% of our consanguineous families with sID [37]. Previously Kahrizi et al. and Mercan et al. have reported de novo variants in approximately 17% and 28% of the individuals with ID born to consanguineous parents, respectively, thus highlighting the significant occurrence of de novo events within highly inbred populations [38, 39].

There are few limitations of our study. Though we report a high rate of identifying de novo disease-causing variants underlying sIDs using a singleton ES in the known disease-causing genes, only one proband with new disease gene association could be ascertained [15]. This could be explained by an inability to perform trio ES or GS in the undiagnosed families due to resource limitations. Also, further investigation may be needed for individuals with undiagnosed phenotypes, such as exploring variants beyond the exonic regions, somatic alterations, digenic as well as oligogenic etiologies.

The knowledge of rare genetic disorders, their diagnoses using the rapidly emerging genomic testing techniques, genetic counseling, prenatal testing, early intervention, and management in LMICs is improving owing to the availability of better infrastructure, cost effective genomic testing, manpower and trained professionals [40, 41]. We herein consolidate the phenotypic and genotypic spectrum of de novo variants underlying monogenic sIDs highlighting the utility of singleton ES as an excellent diagnostic tool for the diagnosis of heterogeneous sIDs in LMIC like India. Our study reiterates de novo variants are likely to contribute significantly as the most frequent cause of sIDs even in populations with consanguinity and endogamy.

## Supplementary Material

Supplementary File

Supplementary Table S1

## Figures and Tables

**Table 1:** Clinical and genotypic details of 54 families (55 individuals) with de novo variants in our cohort.

ID		Age	Gender	Geneticfloat diagnosisfloat attained by	Disorder (MIM)	Inheritance pattern		Gene and variant nomenclature	ACMGfloat classification
Single nucleotide variants and indels									
P1		8 M	Male	Trio ES	Intellectual developmental disorder,float X-linked 93 (300659)^a^	XLR		*BRWD3*float(NM_153252.5): c.828_829dupfloat p.(Lys277IlefsTer24)	Pathogenic
P2		7 Y	Female	Trio ES	Rett syndrome, congenital variantfloat (613454)	AD		*FOXG1*float(NM_005249.5): c.602G>C p.(Arg201Pro)	Likelyfloat pathogenic
P3		3 Y	Male	Singleton ES	Intellectual developmental disorder, X-linked syndromic, Claes-Jensen type (300534)^a^	XLR		*KDM5C*float(NM_004187.5): c.3656T>G p.(Leu1219Arg)	Likelyfloat pathogenic
P4		14 Y	Male	Singleton ES	Neurodevelopmental disorder withfloat coarse facies and mild distal skeletalfloat abnormalities (618505)^a^	AD		*KDM6B*float(NM_001348716.2): c.1603G>Afloat p.(Val535Met)	Likelyfloat pathogenic
P5		6 Y	Female	Singleton ES	Rubinstein-Taybi syndrome 1 (180849)	AD		*CREBBP*float(NM_004380.3): c.1867_1874del p.(Asp623HisfsTer9)	Pathogenic
P6		2 Y	Female	Trio ES	Rett syndrome (312750)	XLD		*MECP2*float(NM_001110792.2): c.352C>T p.(Arg118Trp)	Pathogenic
P7		1 Y	Female	Trio ES	Intellectual developmental disorder,float autosomal dominant 23 (615761)^a^	AD		*SETD5*float(NM_001080517.3): c.1967T>G p.(Leu656Ter)	Pathogenic
P8		12 Y	Female	Singleton ES	Ververi-Brady syndrome (617982)^a^	AD		*QRICH1*float(NM_198880.3): c.1585dupfloat p.(Cys529LeufsTer13)	Pathogenic
P9		6 M	Male	Singleton ES	Cornelia de Lange syndrome 1 (122470)	AD		*NIPBL*float(NM_133433.4): c.328A>T p.(Lys110Ter)	Pathogenic
P10		2 Y	Male	Mendeliome	Tuberous sclerosis-2 (613254)	AD		*TSC2*float(NM_000548.5): c.1096G>T p.(Glu366Ter)	Pathogenic
P11		3 M	Male	Singleton ES	Kabuki syndrome 1 (147920)	AD		*KMT2D* (NM_003482.4): c.6109G>C p.(Asp2037His)	Likelyfloat pathogenic
P12		1 Y	Female	Mendeliome	Lissencephaly, X-linked (300067)^a^	XL		*DCX* (NM_001195553.2): c.536C>A p.(Pro179His)	Likelyfloat pathogenic
P13		1 Y	Male	Singleton ES	Coffin-Siris syndrome 11 (618779)	AD		*SMARCD1* (NM_003076.5): c.1432dupfloat p.(Arg478ProfsTer3)	Pathogenic
P14		1 Y	Female	Trio ES	Bohring-Opitz syndrome (605039)	AD		*ASXL1* (NM_015338.6): c.1435_1436delfloat p.(Pro479ArgfsTer5)	Pathogenic
P15		2 Y	Male	Singleton ES	Megalencephaly-polymicrogyria- polydactyly-hydrocephalus syndrome 2 (615937)	AD		*AKT3* (NM_005465.7): c.1393C>T p.(Arg465Trp)	Pathogenic
P16		3 Y	Male	Singleton ES	Contractural arachnodactyly, congenital (121050)	AD		*FBN2* (NM_001999.4): c.3466T>C p.(Cys1156Arg)	Likely pathogenic
P17		1 Y 6 M	Female	Singleton ES	Craniosynostosis 3 (615314)^a^	AD		*TCF12* (NM_207037.2): c.1807C>T p.(Arg603Trp)	Likely pathogenic
P18		1 Y 6 M	Female	Singleton ES	Neurodevelopmental disorder withfloat absent language and variable seizuresfloat (618707)^a^	AD		*WASF1* (NM_003931.3): c.1516C>T p.(Arg506Ter)	Pathogenic
P19		1 Y	Female	Mendeliome	BRAF-related disorder^a^	AD		*BRAF* (NM_004333.5): c.1406G>A p.(Gly469Glu)	Pathogenic
P20		3 Y	Male	Singleton ES	Coffin-Siris syndrome, 4 (614609)	AD		*SMARCA4* (NM_003072.5): c.3139A>C p.(Asn1047His)	Likely pathogenic
P21		5 Y	Male	Singleton ES	Mucopolysaccharidosis II (309900)	XLR		*IDS* (NM_000202.8): c.305T>C p.(Leu102Pro)	Likely pathogenic
P22		3 Y	Female	Singleton ES	Cortical dysplasia, complex, withfloat other brain malformations 4 (615412)^a^	AD		*TUBG1* (NM_001070.5): c.421A>C p.(lle141 Leu)	Likely pathogenic
P23		1 Y	Male	Singleton ES	Wiedemann-Steiner syndrome (605130)	AD		*KMT2A* (NM_001197104.2: c.5363+1G>A); NC-000011.10: g.118494768G>A	Pathogenic
P24		6 Y	Male	Singleton ES	Sotos syndrome (117550)	AD		*NSD1* (NM_022455.5): c.3017del p.(Pro1006LeufsTer34)	Pathogenic
P25		5 Y	Female	Trio ES	Neurodevelopmental disorder with spastic diplegia and visual defectsfloat (615075)^a^	AD		*CFNNB1* (NM_001904.4): c.1420C>T p.(Arg474Ter)	Pathogenic
P26		5 Y	Male	Trio ES	Intellectual developmental disorder,float autosomal dominant 22 (612337)^a^	AD		*ZBTB18* (NM_205768.3): c.1309C>T p.(His437Tyr)	Likely pathogenic
P27		2 Y	Male	TSS	Mucopolysaccharidosis II (309900)	XLR		*IDS* (NM_000202.8): c.708+2T>C	Pathogenic
P28		2 Y	Female	Singleton ES	Noonan syndrome 8 (615355)	AD		*RIT1* (NM_006912.6): c.170C>G p.(Ala57Gly)	Pathogenic
P29		3 Y	Female	Trio ES	Wiedemann-Steiner syndrome (605130)	AD		*KMT2A* (NM_001197104.2): c.1837C>T p.(Arg613Ter)	Likely pathogenic
P30		6 Y	Male	Trio ES	Intellectual developmental disorder,float autosomal dominant 62 (618793)^a^	AD		*DLG4* (NM_001321075.3): c.1607C>T p.(Pro536Leu)	Likely pathogenic
P31		4 Y	Female	Trio ES	Cleft palate, cardiac defects, andfloat intellectual disability (600987)^a^	AD		*MEIS2* (NM_170675.5): c.973A>G p.(Asn325Asp)	Likely pathogenic
P32		1 Y	Male	Mendeliome	GAND syndrome (615074)^a^	AD		*GATAD2B* (NM_020699.4): c.535C>T p.(Arg 179Ter)	Pathogenic
P33		8 Y	Male	Trio ES	Developmental delay, hypotonia, andfloat impaired language (620012)^a^	AD		*FBXW7* (NM_001349798.2): c.2065C>T p.(Arg689Trp)	Likely pathogenic
P34		5 Y	Female	Singleton ES	Dias-Logan syndrome (617101)^a^	AD		*BCL11A* (NM_022893.4): c.1486G>T p.(Glu496Ter)	Pathogenic
P35		2 Y	Male	Singleton ES	Rett syndrome, congenital variant (613454)	AD		*FOXG1* (NM_005249.5): c.506del p.(G ly 169AlafsTer23)	Pathogenic
P36		10 Y	Male	Mendeliome	Nicolaides-Baraitser syndrome (601358)^a^	AD		*SMARCA2* (NM_003070.5): c.3485G>A p.(Arg1162His)	Likely pathogenic
P37		4 Y	Male	Singleton ES	KBG Syndrome (148050)	AD		*ANKRD11* (NM_001256183.2): c.424C>T p.(Gln142Ter)	Pathogenic
P38		6 Y	Female	Trio ES	Wiedemann-Steiner syndrome (605130)	AD		*KMT2A* (NM_001197104.2): c.4643_4644insACTCCAGGCAAAGG p.(Trp1549Leufs*42)	Pathogenic
P39		4 Y	Male	Singleton ES	Coffin-Siris syndrome 1 (135900)	AD		*ARIDIB* (NM_020732.3): c.3898C>T p.(Gln1300Ter)	Pathogenic
P40		1 Y	Female	Singleton ES	Cortical dysplasia, complex, with otherfloat brain malformations 13 (614563)	AD		*DYNC1H1* (NM_001376.5): c.9751G>A p.(Glu3251 Lys)	Likely pathogenic
P41		3 Y	Female	Singleton ES	Rett syndrome (312750)	XLD		*MECP2* (NM_001110792.2): c.538C>T p.(Arg 180Ter)	Pathogenic
P42		7 Y	Male	Singleton ES	Intellectual developmental disorder,float autosomal dominant 43 (616977)^a^	AD		*HIVEP2* (NM_006734.4): c.5890G>T p.(Gly1964Ter)	Pathogenic
P43		8 Y	Male	Singleton ES	Intellectual developmental disorder,float X-linked 98 (300912)	XLR		*NEXMIF* (NM_001008537.3): c.1441C>T p.(Arg481Ter)	Pathogenic
P44		4 Y	Male	Singleton ES	Intellectual developmental disorder withfloat seizures and language delay (619000)^a^	AD		*SETD1B* (NM_001353345.2): c.4241C>A p.(Ser1414Tyr)	Likely pathogenic
P45		1 Y	Female	Singleton ES	Intellectual developmental disorder,float autosomal dominant 7 (614104)^a^	AD		*DYRK1A* (NM_001347721.2): c.658_659del p.(Met220ValfsTer10)	Pathogenic
P46		9 Y	Male	Singleton ES	Developmental delay, hypotonia,float musculoskeletal defects, and behavioralfloat abnormalities (619595)^a^	AD		*SRCAP* (NM_006662.3): c.6127G>A p.(Gly2043Arg)	Likely pathogenic
P47		3 M	Male	TSS	PCWH syndrome (609136)	AD		*SOX10* (NM_006941.4): c.1400A>T p.(Ter467LeuextTer86)	Likely pathogenic
P48		5 Y	Female	Singleton ES	Intellectual developmental disorder andfloat microcephaly with pontine and cerebellarfloat hypoplasia (300749)	XLD		*CASK* (NM_001367721.1): c.1811del p.(Leu604CysfsTer14)	Pathogenic
P49		1 Y	Male	Singleton ES	MASA syndrome (303350)^a^	XLR		*L1CAM* (NM_001278116.2): c.649A>G p.(Arg217Gly)	Likely pathogenic
P50^b^		9 M	Female	Singleton ES	Bainbridge-Ropers syndrome (615485)^a^	AD		*ASXL3* (NM_030632.3): c.1429dup p.(Ser477PhefsTer2)	Pathogenic
P51^b^		2 Y	Male	Singleton ES	Bainbridge-Ropers syndrome (615485)^a^	AD		*ASXL3* (NM_030632.3): c.1429dup p.(Ser477PhefsTer2)	Pathogenic
P52		8 M	Female	Singleton ES	Noonan syndrome 3 (609942)	AD		*KRAS* (NM_004985.5): c.40G>A p.(Val14lle)	Pathogenic
P53		3 Y	Male	Singleton ES	Rett syndrome, congenital variantfloat (613454)	AD		*FOXG1* (NM_005249.5): c.602G>C p.(Arg201Pro)	Likelyfloat pathogenic
Copy number variants									
P54	1 day		Male	Singleton ES	Rubinstein-Taybi syndrome 1 (180849)		AD	*CREBBP* (NM_004380.3) 16p13.3 (3727580-3851110) x 1	Pathogenic
P55	6 days		Female	Singleton ES	Polycystic kidney disease, infantile severe,float with tuberous sclerosis (600273)		AD	*TSC2-PKD1* 16p13.3 (2069904-2147985) x 1	Pathogenic

*Y* Years, *M* months, *ES* exome sequencing, *TSS* Targeted Sanger sequencing, *AD* Autosomal dominant, *XL* X-linked, *XLR* X-linked recessive, *XLD* X-linked dominant.

a Rare syndromes with ID not reported previously from Indian population.

b P50 and P51 are siblings harboring a de novo variant, c.1429dup p.(Ser477PhefsTer2) in *ASXL3* (NM_030632.3) causing Bainbridge-Ropers syndrome (MIM #615485), suggesting the possibility of gonadal mosaicism in either of the phenotypically unaffected parents.

## Data Availability

Additional data are available from the corresponding author on reasonable request.
