# Supplementary material for: De novo variants underlying monogenic syndromes with intellectual disability in a neurodevelopmental cohort from India
Source: Eur J Hum Genet. 2023 Dec 20;32(10):1291–8. doi: 10.1038/s41431-023-01513-7 (PMC7616498; doi:10.1038/s41431-023-01513-7)
Supplement: Supplementary file 1 — Supplementary material [file 41431_2023_1513_MOESM1_ESM.docx]

***Detailed methodology***

***DNA extraction***

2-5 ml EDTA blood was collected from the proband and their respective parents as available. The genomic DNA was extracted using QIAmp DNA Blood Mini kit and QIAmp DNA Blood Mini kit (QIAGEN, Hilden, Germany) following the standard protocols.

***Chromosomal microarray and analysis of copy number variants***

Chromosomal microarray (CMA) was done in 48 individuals with multiple congenital anomalies with intellectual disability with developmental delay or autism as a first tier of testing. CMA was performed using the Affymetrix CytoScan 750K array (Santa Clara, California) and the resulting data was analysed using Chromosome Analysis Suite (ChAS) v.4.2.1; Illumina’s Infinium Global Screening Array BeadChip (San Diego, California) and the data was analysed using the KaryoStudio v1.4; Genomestudio v2.0 and Agilent G5923A Sureprint G3 Human CGH array (Santa Clara, California) with the data analysis performed using the software, Cytogenomics v5.2. The details are provided in supplementary table S2.

Publicly available database like the Database of Genomic variants (DGV) and Database of chromosomal imbalances and phenotypes in human using ensemble resources (DECIPHER), gnomAD, ClinVar and the published literature were used to study the CNVs identified in the affected individuals. Briefly, the CNVs were evaluated for being rare or commonly reported in the population; presence of protein coding or morbid genes in the CNV interval were critically evaluated for their disease association, co-relation with patient phenotypes, the disease mechanism and inheritance pattern. The significance of the identified CNVs in an individual were then classified into pathogenic, likely pathogenic, variant of uncertain significance, likely benign or benign based on American College of Medical Genetics and Genomics (ACMG) guidelines and ClinGen criteria.

***Exome sequencing***

Based upon the clinical presentation and family history, a singleton exome sequencing was performed in 50 families, a duo exome sequencing of two affected individuals in the family was performed in six families and a proband parents trio exome sequencing was performed in 15 families. Exome sequencing (ES) was performed using standard protocols (1). The NGS platforms, capture kits and read depth details used for exome sequencing in the cohort are given in supplementary table S1. The first step was filtering the variants with minor allele frequency of >1% in population database like gnomAD (2), SG100K (3), genomeAsia (4) and our in-house data of 2950 exomes comprised of affected individulas with rare mendelian disorders and their unaffected parents (5). The next step was prioritization based upon the location of the variant, intergenic, and deep intronic variants are excluded as it is believed that at least 80% of the disease-causing variants underlying the monogenic disorder lie in the protein coding or the exonic region of the genome (6). Variants were then segregated based on zygosity (homozygous, heterozygous, compound heterozygous and hemizygous). In case of a parent-child trio or quad ES, variants that did not segregate were excluded while in duo sequencing for two similarly affected individuals, variants segregating in both were prioritized. The remaining variants were prioritized based on concordance with phenotype observed in the patient with Online Mendelian Inheritance in Man (OMIM) phenotypes and Human Phenotype Ontology (HPO) terms and multiple lines of *in silico* pathogenicity predictions. Sanger sequencing was performed as necessary either for segregation analysis or for validating the same.

**Mendeliome**

Based upon the provisional clinical diagnosis of a known monogenic disorder, a Mendeliome or targeted exome sequencing was performed in five families with achievement of a molecular diagnosis in all the families. A Mendeliome involved next generation sequencing of exons of 6670 genes using the Clinical Exome version 4 capture kit (MedGenome Labs. Pvt Ltd.). The processing, annotating, filtering and analyzing strategies were same as that for exome sequencing as described above.

***Copy number variant analysis from exome sequencing data***

CNV analysis from the exome sequencing data was carried out in patients where exome analysis did not reveal any significant variant. Briefly, “hidden Markov model (XHMM)”, “ExomeDepth” and “cn.mops” protocol was followed for CNV calling from the binary alignment file . CNVs were called against ES data of capture-kit matched reference samples from our in-house dataset (CREv2, CREv3, CEV4 and TWIST). CNVs in genomic regions/genes previously implicated in a specific multiple congenital anomaly syndrome or disease were filtered for any significant variations along with its rarity. The retrieved CNV’s were then filtered using Database of Genomic Variants (DGV) and gnomAD or Database of Chromosomal Imbalance and Phenotype in Humans Using Ensemble Resources (DECIPHER) and ClinVar to elucidate its presence in population or in affected individuals respectively. The CNV interval was looked for significant genes for concordance with inheritance pattern, disease mechanism, phenotypes associated from the literature in addition to referring to the currently evolving CNV interpretation tools like ClinGen (7). The called CNVs were then validated using a quantitative PCR (qPCR) using the absolute quantification principle or a chromosomal microarray.

**Supplementary table S2**

| **Platform** | **Type of**  **reads** | **Capture kit** | **Target**  **region** | **Sequencing**  **coverage at 10X** |
| --- | --- | --- | --- | --- |
| NovaSeq6000 | PE 2x150 | Agilent SureSelect Clinical Research Exome v2 (Santa Clara, California) | 67mb | >95% |
| NovaSeq6000 | PE 2x150 | Agilent SureSelect Clinical Research Exome v3 (Santa Clara, California) | 70mb | >95% |
| NovaSeq6000 | PE 2x150 | Clinical Exome version 4 | 50mb | >95% |
| NovaSeq6000 | PE 2x150 | TWIST and a modified TWIST (South San Francisco California) | 50mb | >95% |

**Table S2**. NGS platforms, capture kit and read depth details used for exome sequencing in the cohort.

**Supplementary table S3**

| **S no** | **Microarray platform** | **Analysis software** |
| --- | --- | --- |
| 1 | Affymetrix CytoScan 750K array (Santa Clara, California) | Chromosome Analysis Suite (ChAS) v4.2 |
| 2 | Illumina’s Infinium Global Screening Array BeadChip (San Diego, California) | KaryoStudio v1.4; Genomestudio v2.0 |
| 3 | Agilent G5923A Sureprint G3 Human CGH array (Santa Clara, California) | Cytogenomics v5.2 |

**Table S3.** Chromosomal microarray platforms and analysis software

**Supplementary figure S1**


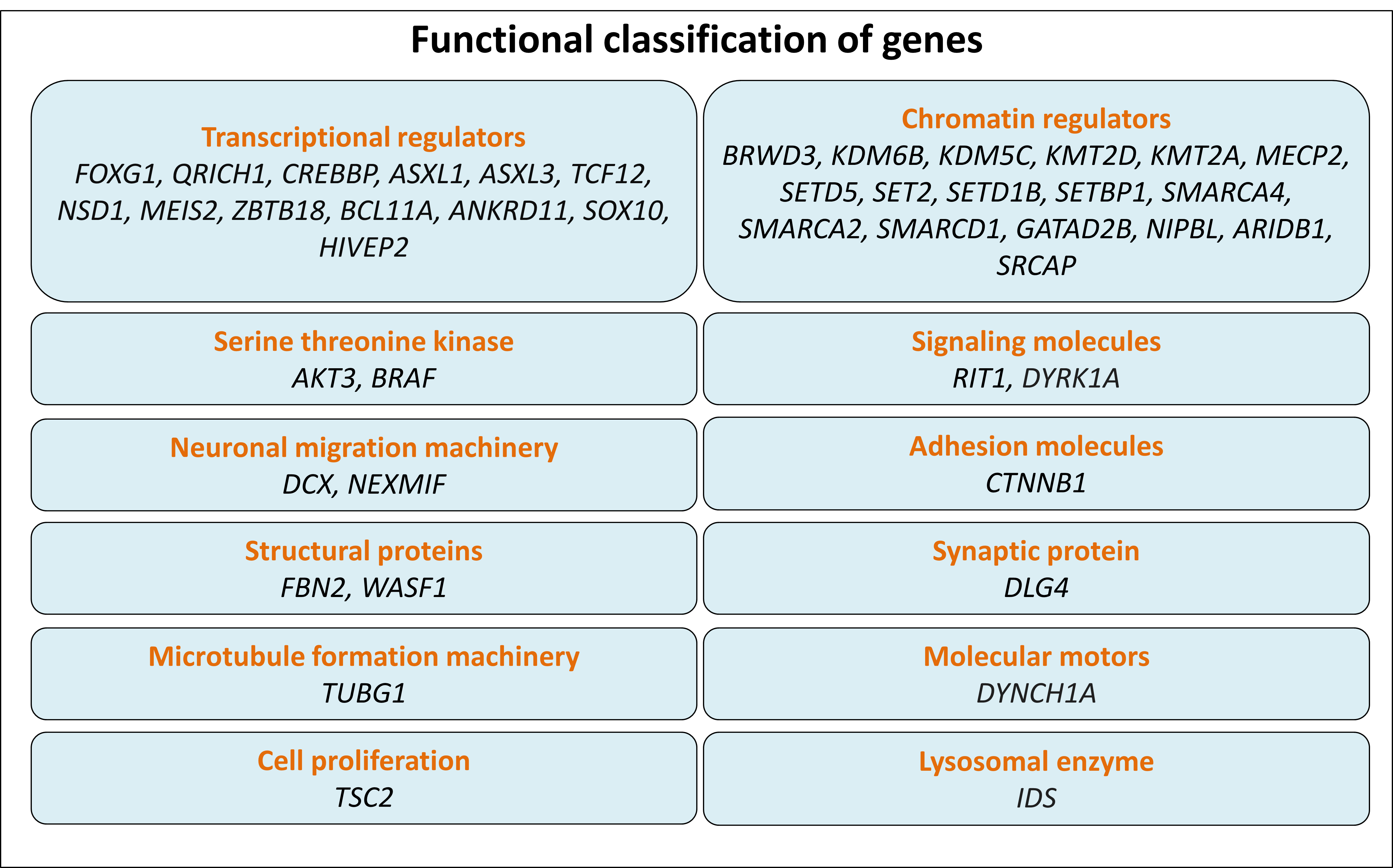


**Figure S1.** Functional classification of genes identified in the cohort.

1. Kaur P, do Rosario MC, Hebbar M, Sharma S, Kausthubham N, Nair K, et al. Clinical and genetic spectrum of 104 Indian families with central nervous system white matter abnormalities. Clin Genet. 2021;100(5):542-50.

2. Lek M, Karczewski KJ, Minikel EV, Samocha KE, Banks E, Fennell T, et al. Analysis of protein-coding genetic variation in 60,706 humans. Nature. 2016;536(7616):285-91.

3. Wu D, Dou J, Chai X, Bellis C, Wilm A, Shih CC, et al. Large-Scale Whole-Genome Sequencing of Three Diverse Asian Populations in Singapore. Cell. 2019;179(3):736-49.e15.

4. Wall JD, Stawiski EW, Ratan A, Kim HL, Kim C, Gupta R, et al. The GenomeAsia 100K Project enables genetic discoveries across Asia. Nature. 2019;576(7785):106-11.

5. Kausthubham N, Shukla A, Gupta N, Bhavani GS, Kulshrestha S, Das Bhowmik A, et al. A data set of variants derived from 1455 clinical and research exomes is efficient in variant prioritization for early-onset monogenic disorders in Indians. Hum Mutat. 2021;42(4):e15-e61.

6. Lupski JR, Belmont JW, Boerwinkle E, Gibbs RA. Clan genomics and the complex architecture of human disease. Cell. 2011;147(1):32-43.

7. Rehm HL, Berg JS, Brooks LD, Bustamante CD, Evans JP, Landrum MJ, et al. ClinGen--the Clinical Genome Resource. N Engl J Med. 2015;372(23):2235-42.
